# Development and validation of a nomogram incorporating OSA, age, and BMI to predict cognitive impairment after acute mild ischemic stroke

**DOI:** 10.1097/MD.0000000000048201

**Published:** 2026-04-10

**Authors:** Huiting Wang, Guifeng Zhang, Zheng Wang, Jingjing Zhang

**Affiliations:** a Department of Neurology, Liaocheng People’s Hospital, Liaocheng, Shandong, China; b Department of Neurosurgery, Liaocheng Traditional Chinese Medicine Hospital, Liaocheng, Shandong, China.

**Keywords:** apnea hypopnea index, cognitive dysfunction, mild ischemic stroke, nomogram, obstructive sleep apnea

## Abstract

This study used a nomogram to predict cognitive impairment in acute mild ischemic stroke patients. This study retrospectively included 248 patients with acute mild ischemic stroke admitted to the Department of Neurology, Liaocheng People’s Hospital, between October 31, 2022, and September 10, 2023. Cognitive impairment was defined using the Mini-Mental State Examination. The total sample was divided using a split-sample approach into a training set (n = 174) and a validation set (n = 74). We examined clinical and general data to identify cognitive dysfunction risk factors. Multivariate logistic regression identified obstructive sleep apnea (OSA), age, and body mass index (BMI) as independent risk factors for cognitive impairment. These factors were integrated to develop the nomogram as an early warning model. The predictive model was validated internally (training set) and externally (validation set). Its discriminative ability was assessed using receiver operating characteristic curves and its accuracy using calibration curves. The model demonstrated robust predictive performance in both the training set (area under the curve (AUC) = 0.808) and the validation set (AUC = 0.733). The calibration curves indicated good agreement between the predicted probability and the observed outcomes. Overall, the developed nomogram, which integrates OSA, age, and BMI, serves as a simple and effective early warning tool for predicting cognitive impairment in patients with acute mild ischemic stroke, offering valuable guidance for clinical intervention.

## 1. Introduction

Acute ischemic stroke is a prevalent cerebrovascular condition that exerts a significant influence on the patient’s physical well-being, while also potentially resulting in cognitive decline and other neuropsychological consequences.^[1]^ Based on statistical data, it has been shown that approximately 33% of individuals who have experienced a stroke will manifest cognitive impairment, with approximately 50% of these individuals experiencing long-term or permanent cognitive impairment.^[2,3]^ The patient’s life, social functioning, and mental health are greatly affected by this cognitive disability.^[4,5]^ Nevertheless, there is a dearth of risk prediction tools for post-acute ischemic cognitive impairment, underscoring the significance of creating efficient prediction models and implementing suitable early therapies. This study identified and demonstrated that the presence of obstructive sleep apnea (OSA), advanced age, and higher body mass index (BMI) are associated with an elevated likelihood of cognitive impairment following an acute mild ischemic stroke. Beyond traditional vascular risk factors, OSA, as a highly prevalent yet often underestimated comorbidity, exerts a critical influence on cognitive outcomes in patients with ischemic stroke.^[6]^ Epidemiological studies show that the prevalence of OSA in stroke patients is exceedingly high, several times that of the general population.^[7]^ This high burden establishes OSA as an important and potentially modifiable risk factor for predicting post-stroke cognitive impairment. The key mechanism by which OSA contributes to PSCI is intermittent hypoxia (IH). IH induces recurrent hypoxia-reoxygenation cycles, leading to systemic inflammatory responses and oxidative stress.^[8]^ These pathological changes compromise vascular endothelial function, accelerating atherosclerosis and cerebral small vessel disease.^[9]^ Ultimately, this chronic hypoxia-inflammation state exacerbates post-stroke ischemic brain injury and renders neurons more vulnerable to secondary damage, which accelerates cognitive decline.^[10]^ Given the high prevalence of OSA in the stroke population and its direct detrimental effect on cognitive function, its inclusion in the predictive model holds significant clinical value. Additionally, a scoring tool based on a nomogram was developed to forecast the occurrence of cognitive impairment following acute mild ischemic stroke.

## 2. Materials and methods

### 2.1. Object of study

The collection of data took place between October 31, 2022 to September 10, 2023 inside the Department of Neurology at Liaocheng People’s Hospital. The research encompassed a total of 248 individuals diagnosed with acute mild ischemic stroke. These individuals were assigned to either a training group consisting of 174 patients or a validation group consisting of 74 patients, employing the leave-out technique. The study adhered to the pertinent sections of the Declaration of Helsinki, received approval from the Ethics Committee of Liaocheng People’s Hospital associated with Shandong First Medical University (Approval No: 2024019). The data for this study were obtained from electronic case information of previously hospitalized patients, and as a retrospective study, informed consent waivers were available.

### 2.2. Inclusion criteria

Patients with acute ischemic stroke admitted to the Department of Neurology of Liaocheng People’s Hospital from August 2022 to May 2023 The inclusion criteria were as follows:

1. Eighteen to 75 years of age;
2. Diagnosis of acute ischemic stroke confirmed by 2 experienced neurologists through computed tomography and/or magnetic resonance imaging; first diagnosis of cerebral infarction; within 7 days of onset.
3. NIHSS score of 1 to 5 on admission, defined as mild ischemic stroke;
4. First diagnosis of cerebral infarction; within 7 days of onset;
5. Acceptance to participate in all tests.

### 2.3. Exclusion criteria

1. Respiratory failure and advanced congestive heart failure.
2. Any preexisting cognitive impairment or psychiatric co-morbidity prior to the occurrence of a stroke.
3. The conditions under consideration include central sleep apnea, complicated sleep apnea, or a previous diagnosis of OSA.
4. Inability to undergo all relevant examinations and tests.

### 2.4. The process of gathering general information

The researcher collected general demographic data, such as age, gender, and occupation, for both the cognitively impaired and non-cognitively impaired groups. Additionally, the researcher recorded the individuals’ height, weight level, years of education, smoking status, and alcohol use. The smoking status was classified into 2 groups: smokers and nonsmokers. Similarly, the drinking status was classified into 2 groups: drinkers and nondrinkers, depending on their prior alcohol intake. The BMI was determined by dividing the weight (kg) by the height (m).

### 2.5. A critical evaluation of obstructive sleep apnea

The portable sleep apnea monitoring device was utilized to assess OSA in each participant. The AHI, or apnea hypopnea index, was calculated as the average number of apneas and hypopneas per hour of sleep. Patients with an AHI ≤ 5 were clinically diagnosed with OSA.

### 2.6. Cognitive assessment

The Mini-Mental State Examination (MMSE) is a commonly employed assessment tool on a global scale for evaluating cognitive abilities.^[11]^ Enrolled patients underwent standardized neuropsychological assessments within 7 days following an acute stroke. All tests were conducted in a sound and isolated room. The patients in the training and validation sets were categorized into cognitively impaired and non-cognitively impaired groups based on their MMSE scores.

### 2.7. statistical analysis

R 4.1.2 was used to handle and analyze the data. Count data were described by case percentage, and group comparisons used the chi-square test. Missing data analysis showed minimal missingness. Due to the minimal missing data for BMI (3/174 patients, <2%), Listwise Deletion was employed. Potential influence factors were first determined using univariate logistic regression. Multifactorial logistic regression was then performed on variables that were statistically significant in the univariate analysis (*P* < .05). Collinearity among the final predictors (OSA, age, and BMI) was assessed using the variance inflation factor; all variance inflation factor values were below 4 confirming no significant collinearity. The final predictive model, a nomogram, was constructed based on the multifactorial analysis results. Model validation was performed using Bootstrap validation (1000 repetitions) for internal validation. The overall procedures for nomogram construction and validation were strictly adhered to established methodological guidelines.^[12,13]^ Decision curve analysis was utilized to evaluate the model’s clinical net benefit. *P*-values below .05 were considered statistically significant.

## 3. Result

### 3.1. Clinical characteristics analysis

The study included 248 participants, with a mean age of 62.58 ± 11.063 years, consisting of 146 (58.87%) males and 102 (41.13%) females. 70% were randomized to development, and the rest to validation. No significant difference was seen in cognitive impairment, OSA occurrence, gender, age, BMI, education level, smoking, or alcohol intake between the 2 groups, *P* > .05. Refer to Table 1.

**Table 1:** Development group validation group clinical and demographic data.

Characteristics	Development group	Validation group	Statistic	*P* value
Number	174	74		
Cognitive dysfunction, n (%)				
Yes	39 (15.7%)	17 (6.9%)	0.009	.923
No	135 (54.4%)	57 (23%)		
OSA, n (%)				
Yes	110 (44.4%)	43 (17.3%)	0.574	.449
No	64 (25.8%)	31 (12.5%)		
Gender, n (%)				
Male	99 (39.9%)	47 (19%)	0.939	.333
Female	75 (30.2%)	27 (10.9%)		
Age, mean ± SD	62.776 ± 11.603	62.122 ± 9.732	0.4259	.671
BMI, mean ± SD	25.872 ± 3.7374	26.322 ± 6.8407	–0.66	.51
Years of education, mean ± SD	5.0805 ± 3.4431	5.0676 ± 3.1724	0.028	.978
Smoking, n (%)				
Yes	74 (29.8%)	38 (15.3%)	1.631	.201
No	100 (40.3%)	36 (14.5%)		
Alcohol drinking, n (%)				
Yes	72 (29%)	32 (12.9%)	0.074	.785
No	102 (41.1%)	42 (16.9%)		

BMI = body mass index, OSA = obstructive sleep apnea, SD = standard deviation.

### 3.2. Analysis of acute mild ischemic stroke patients’ cognitive impairment risk factors

The study examined 7 potential predictors of cognitive dysfunction: OSA, age, gender, BMI, years of schooling, smoking, and alcohol intake. Univariate and multivariate regression analyses revealed that OSA, age, and BMI were significant independent risk factors for cognitive dysfunction in acute mild ischemic stroke patients. Specifically, patients with OSA showed a significantly increased risk (odds ratio (OR) = 4.946, 95% confidence interval (CI): 1.667–14.176, *P* = .004). Each additional year of age was associated with an increased risk (OR = 1.088, 95% CI: 1.038–1.141, *P* < .001). Furthermore, a one-unit increase in BMI was associated with an increased illness risk (OR = 1.234, 95% CI: 1.089–1.397, *P* = .001). Refer to Table 2.

**Table 2:** Univariate multifactor regression analysis.

Characteristics	Total (n)	Univariate analysis		Multivariate analysis	
		Odds ratio (95% CI)	*P* value	Odds ratio (95% CI)	*P* value
OSA, n (%)	174				
No	71	Reference		Reference	
Yes	103	5.279 (1.945–14.328)	.001	4.946 (1.667–14.176)	.004
Gender, n (%)	174		.304		
0	72	Reference			
1	102	0.679 (0.325–1.419)			
Age, mean ± SD	174	1.05 (1.012–1.088)	.009	1.088 (1.038–1.141)	<.001
BMI, mean ± SD	171	1.181 (1.066–1.308)	.001	1.234 (1.089–1.397)	.001
Years of education, mean ± SD	174	0.934 (0.844–1.038)	.203		
Smoking, n (%)	174		.879		
Yes	94	Reference			
No	80	1.057 (0.515–2.171)			
Alcohol drinking, n (%)	174				
Yes	100	Reference			
No	74	0.52 (0.253–1.068)	.075		

BMI = body mass index, OSA = obstructive sleep apnea, SD = standard deviation.

### 3.3. Development and validation of the nomogram

**Figure 1.**
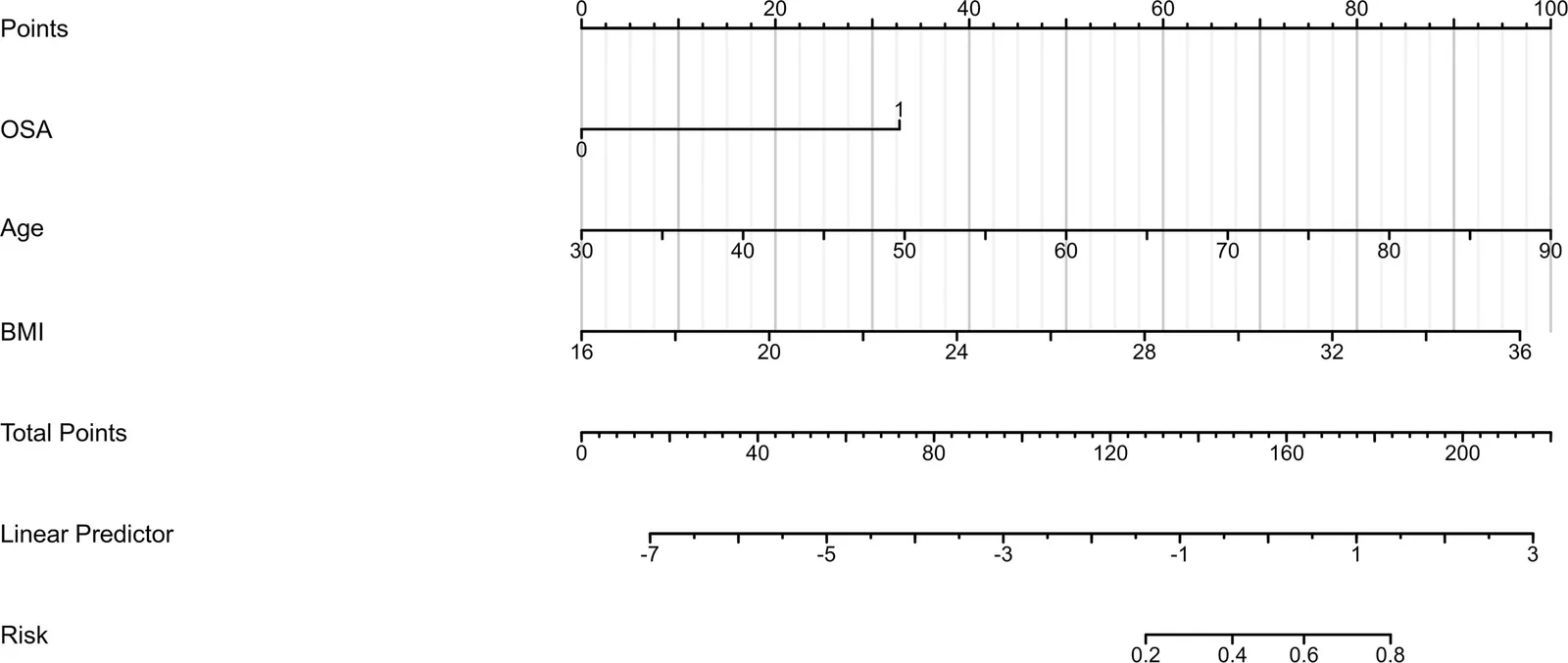
Nomogram for forecasting the likelihood of cognitive impairment in patients with acute mild ischemic stroke. This scoring tool integrates obstructive sleep apnea (OSA), age, and body mass index (BMI). By summing the scores corresponding to the variables on the “points” scale, the individual’s total score can be projected onto the “total score” scale to determine the predicted probability of cognitive impairment.

**Figure 2.**
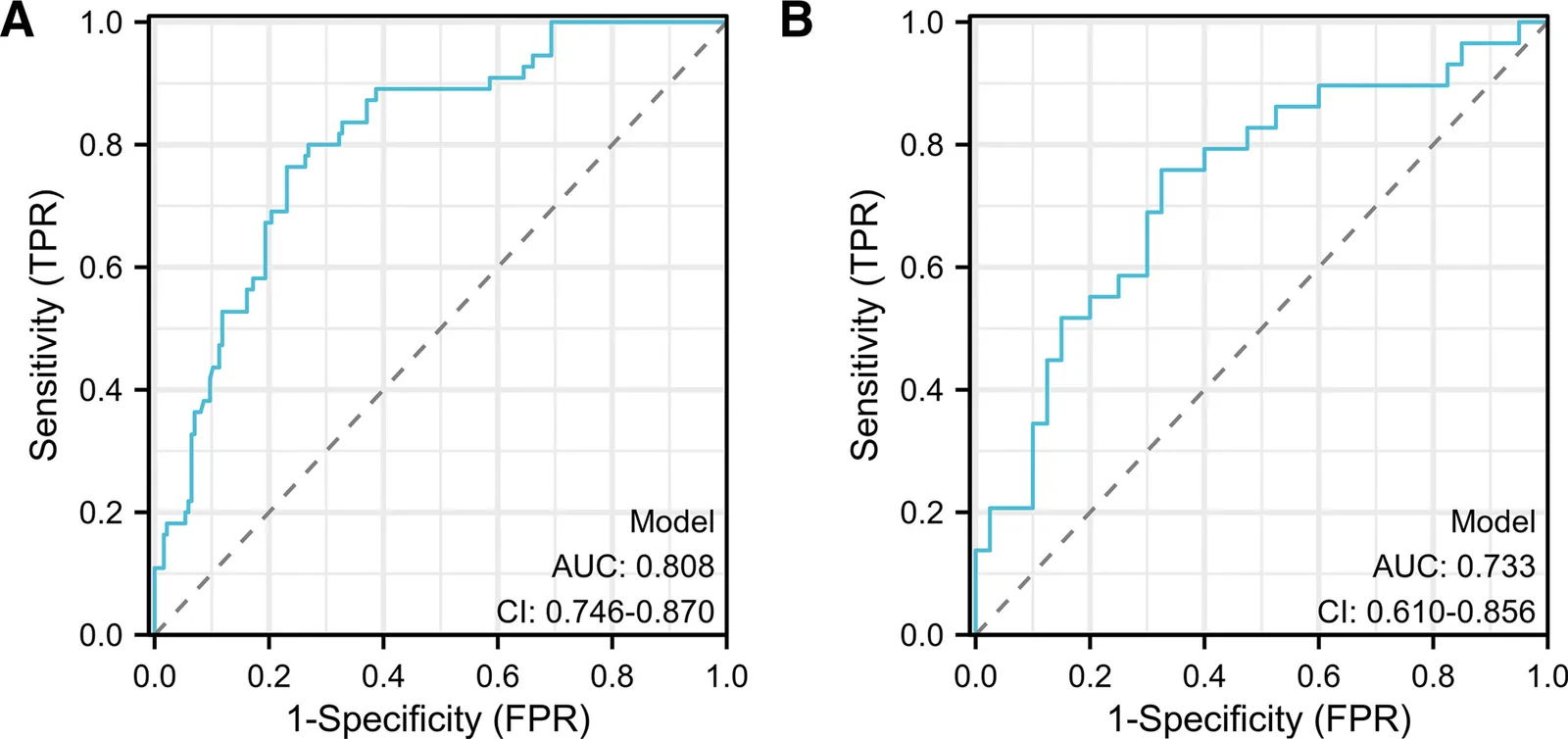
Receiver operating characteristic (ROC) curves of the nomogram. (A) ROC curve for the training group (internal validation). The blue line represents the predictive performance of the model, and the gray dashed line represents the diagonal reference line (AUC = 0.5), indicating no discriminative ability. (B) ROC curve for the validation group (external validation).

**Figure 3.**
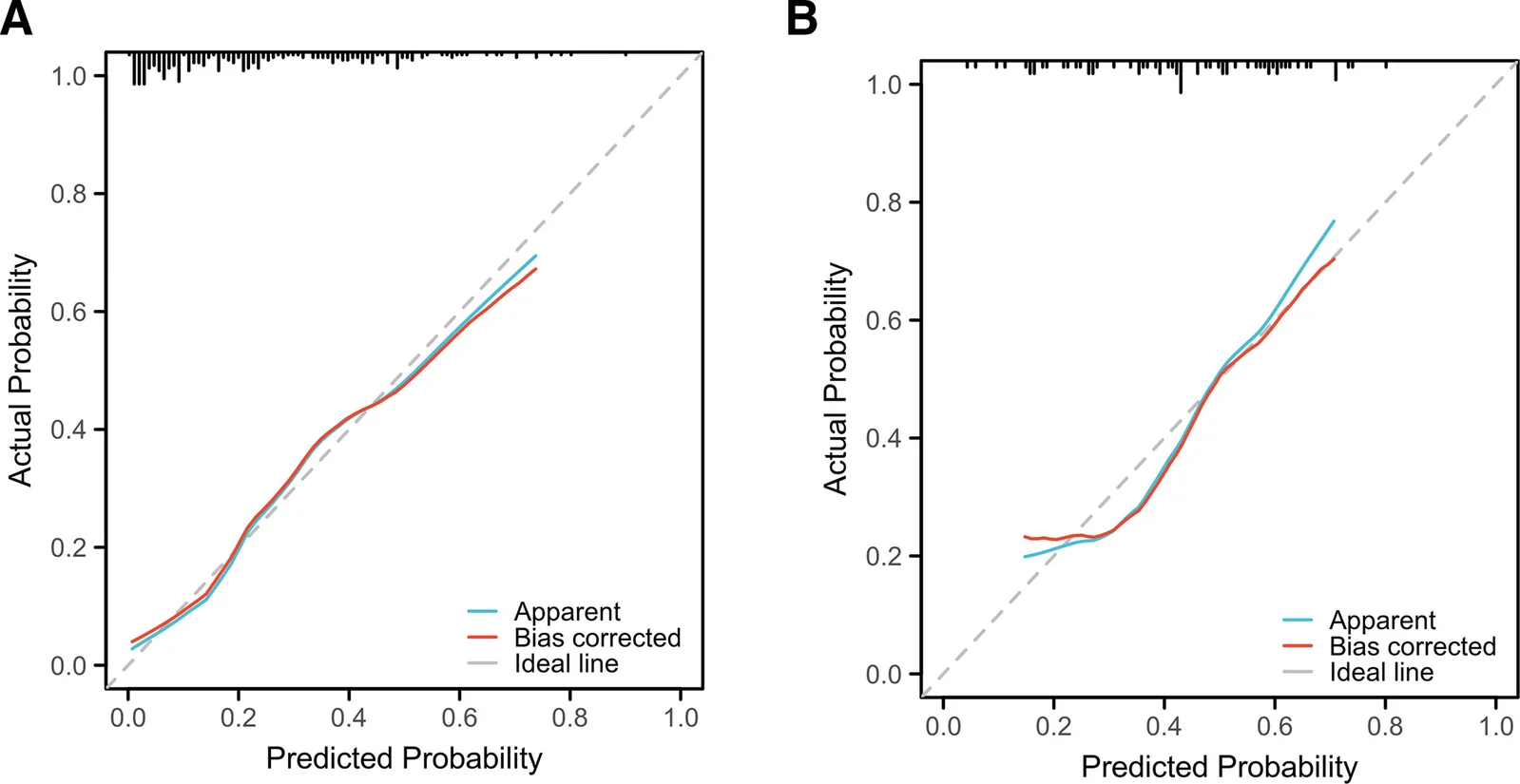
Calibration curves of the nomogram. (A) Calibration curve for the development (training) set. The blue line represents the model’s predicted probability, and the gray dashed line represents the ideal reference line, where the predicted probability perfectly matches the observed rate. (B) Calibration curve for the validation set.

The OSA age BMI was utilized to create a nomogram, where each variable represented a score. The scores for the 3 variables contained in the model were then combined to get the individual’s overall score. The probability of cognitive dysfunction was determined by projecting the individual’s total score onto the total score scale, as depicted in Figure 1. The area under the curve values for the development and validation sets were 0.808 (95% CI: 0.746–0.870) (Fig. 2A) and 0.733 (95% CI: 0.610–0.859) (Fig. 2B), respectively. Both the training set (Fig. 3A) and validation set (Fig. 3B) exhibited a high level of agreement between the predicted and observed rates.

## 4. Discussion

The present study found that individuals with combined OSA had a 4.279-fold increased risk of cognitive impairment compared to those without moderate OSA. This finding aligns with prior research, indicating that OSA is linked to repetitive apnea, chronic hypoxia, oxygen desaturation, and hypercarbia.^[14–16]^ Furthermore, these IH conditions have the potential to elicit an inflammatory response, resulting in neuronal damage, specifically in the hippocampus and cortex.^[17–19]^

Consistent with previous studies, the present study shows that age is a risk factor for the development of cognitive dysfunction in patients with acute stroke.^[19–21]^ It is widely recognized that with advancing age, cerebral blood arteries undergo a slow process of arterial stiffening, characterized by thickening walls and reduced elasticity.^[22]^ These physiological changes can potentially result in impaired cerebrovascular function.^[23]^ The decline in cerebrovascular function can lead to ischemic damage, which can result in conditions such as cerebral infarction or cerebral thrombosis, thereby significantly affecting the blood flow to brain tissue.^[24–26]^ The concept of collateral circulation pertains to the occurrence wherein the obstruction of major blood arteries prompts the compensatory expansion and proliferation of minor blood vessels to compensate for the deficiency in blood flow.^[27–29]^ The decline in collateral circulation with advancing age is attributed to the diminished functionality of vascular endothelial cells, reduced proliferative capacity of vascular smooth muscle cells, and decreased angiogenic factor. Consequently, these factors exacerbate the severity of cerebral ischemia and hypoxia.^[30,31]^ Extended hypoxia-ischemia leads to significant harm to brain tissue, leading to neuronal demise, diminished synapses, and compromised neurotransmitter production.^[10,32]^ These pathological alterations can have a detrimental impact on cognitive abilities, including memory impairment, reduced cognitive speed, and impaired focus.^[32]^ Animal tests have demonstrated that enhancing cerebrovascular function or facilitating the formation of collateral circulation can significantly mitigate cerebral ischemia-hypoxia injury, hence enhancing cognitive function.^[33]^ Hence, to mitigate and enhance cognitive decline, it is imperative to prioritize the preservation of cerebrovascular well-being and implement proactive strategies to foster the development of collateral circulation^.[34]^ These strategies encompass adopting a healthy lifestyle, engaging in suitable physical activity, and effectively managing blood pressure and blood glucose levels.

Higher BMI is associated with cognitive dysfunction in ischemic stroke patients, according to this study.^[35]^ Previous research on BMI and cognitive performance has found mixed results. Some studies show that elevated BMI decreases cognitive function, while others suggest that it protects it.^[36]^ BMI affects cognitive performance depending on age, according to research. BMI protects cognitive function in those under 45 but negatively affects it in people 45 and older. However, few studies have examined the relationship between BMI and cognitive performance in acute ischemic stroke patients. This study found a positive link between age and cognitive deterioration in acute ischemic stroke patients. Higher BMI promotes plaque development, and atherosclerosis reduces cerebral blood flow.^[36–38]^ Thus, stroke patients’ higher ischemia and hypoxia increase cognitive decline risk. An increased risk of cognitive decline.^[32]^

Following extensive investigation and analysis, the primary factors used for the prediction model in this study were age, BMI, and sleep apnea disorder. The consideration of age is crucial due to the progressive decline of several physiological systems in the human body, including cognitive function.^[39]^ Additionally, the BMI serves as an indicator of patients’ nutritional status and obesity level, which is strongly associated with the likelihood of experiencing cognitive impairment.^[35]^ In contrast, sleep apnea has the potential to induce cerebral hypoxia, hence exacerbating the impact on cognitive performance.^[40]^ The utilization of this prognostic model would significantly enhance the timely detection of cognitive decline following an acute stroke, empowering medical practitioners to intervene earlier in order to facilitate cognitive recovery and enhance patients’ overall well-being. Simultaneously, the model will serve as a potent instrument for forthcoming cognitive impairment research and foster the advancement of associated domains. Throughout the model’s building, we employed sophisticated statistical techniques and machine learning algorithms to guarantee the model’s precision and dependability. By validating a substantial volume of clinical data, we have determined that the model can precisely forecast the likelihood of cognitive impairment in patients suffering from acute ischemic stroke. This offers clinicians robust decision-making assistance.

Nomograms have the ability to visually represent the likelihood of illness incidence using straightforward graphs. These charts are highly intuitive, uncomplicated, and convenient to utilize, making them extensively employed in the field of medical research.^[41,42]^ We conducted univariate and subsequent multivariate Cox regression analysis to identify OSA, age, and BMI as risk factors for cognitive failure in mild ischemic stroke. Based on these risk variables, we developed a predictive model using a nomogram. The internal and external validation, as well as the receiver operating characteristic curves, of the training and validation sets indicate that the model exhibits a high level of accuracy and dependability. Moreover, it has potential clinical applicability. This study is the inaugural attempt to construct a nomogram of cognitive impairment in mild acute ischemic stroke patients who exhibit OSA. While this approach is somewhat innovative, it is important to note that the study’s clinical sample size is insufficient, which may introduce some bias. Consequently, in subsequent phases, a greater number of patients will be documented in order to enhance the existing nomogram.

## 5. Conclusion

Ultimately, the predictive model developed in this study, which takes into account age, BMI, and sleep breathing condition, holds significant therapeutic significance. This intervention will offer robust assistance in evaluating the likelihood of cognitive decline in individuals suffering from acute ischemic stroke, facilitate timely intervention and recovery, and contribute to the holistic rehabilitation of stroke patients.

## Acknowledgments

The authors would like to thank all patients and medical staff at the Department of Neurology, Liaocheng People’s Hospital, for their cooperation and support in this study.

## Author contributions

**Conceptualization:** Huiting Wang.

**Data curation:** Guifeng Zhang, Zheng Wang.

**Funding acquisition:** Huiting Wang.

**Visualization:** Guifeng Zhang, Zheng Wang.

**Writing – original draft:** Huiting Wang, Guifeng Zhang.

**Writing – review & editing:** Huiting Wang, Jingjing Zhang.
